# Preservation of genetic diversity in a highly fragmented population of the gray‐sided vole *Myodes rufocanus* in an intensive farming region

**DOI:** 10.1002/ece3.10472

**Published:** 2023-09-19

**Authors:** Yasuyuki Ishibashi

**Affiliations:** ^1^ Hokkaido Research Center Forestry and Forest Products Research Institute Sapporo Japan

**Keywords:** fragmented habitats, gene flow, genetic diversity, male‐biased dispersal, microsatellite DNA, mtDNA

## Abstract

Individual dispersal plays an important role in preserving genetic diversity in density‐fluctuating populations of arvicoline rodents. When habitats are fragmented and dispersal between habitats is severely constrained, genetic diversity can be lost. Here, I investigated whether genetic diversity in the gray‐sided vole *Myodes rufocanus* was preserved in an intensive farming region in Japan, where voles inhabited isolated windbreak forests along the borders of plowed lands. Genetic structure was examined in 673 vole samples (330 in spring and 343 in fall) collected at 34 windbreak forests located 0.35–20 km apart. A part of the control region (425 bp) of mitochondrial DNA (mtDNA) was sequenced in 673 voles, yielding 76 haplotypes. Genetic differentiation of maternally inherited mtDNA among trapping sites was markedly lower in males than in females in both seasons, indicating strong male‐biased dispersal. Genotypes at six microsatellite DNA loci were determined in 494 voles (245 in spring and 249 in fall) from 18 trapping sites, and loci harbored 16–24 alleles. The mean number of alleles per locus (allelic diversity) at trapping sites was positively correlated with the number of examined individuals (density) in both seasons, and the relationship was very similar to that of a previous study performed in much less fragmented populations. The genetic differentiation of microsatellite DNA among trapping sites decreased considerably from spring to fall. In a STRUCTURE analysis with a most probable cluster number of two, closer trapping sites showed more similar mean values of cluster admixture proportions. The present findings indicate that gene flow among isolated windbreak forests, which occurred mainly by dispersal of males, was not restrained in this intensive farming region. Furthermore, the results suggest that genetic diversity in the study population was preserved as well as in less fragmented populations.

## Graphical abstract

In the STRUCTURE analysis, admixture cluster proportions were spatially heterogeneous, and closer trapping sites showed more similar mean values of cluster admixture. Females and males were consistent in terms of the directions of changes in the mean admixture proportions between spring (left) and fall (right) at each trapping site, although the degree of change in males was greater than in females at most sites.

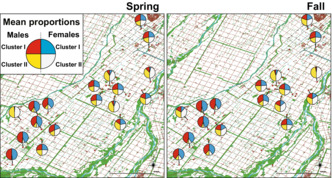

## 1 INTRODUCTION

Drastic human‐induced changes to landscapes are taking place worldwide, causing fragmentation of natural and seminatural habitats. Fragmentation results in an altered configuration of habitats with decreasing size and increasing average distance between habitats. For animals with low dispersal ability, even separation of a short distance may interfere with individual dispersal between fragmented habitats. Without individual dispersal, a loss of genetic variation (genetic erosion) could occur by genetic drift and inbreeding in small, isolated subpopulations, and the diminished genetic variation could reduce the opportunity for adaptive responses to varying local environmental conditions (Frankham et al., 2017; Sherwin & Moritz, 2000). Furthermore, decreased genetic variation within subpopulations may result in the reduced viability of the entire population due to low reproductive and survival rates (Sherwin & Moritz, 2000).

Arvicoline rodents often undergo 3–5‐year‐cycle fluctuations of 10–100‐fold changes in density (Stenseth, 1999). Genetic diversity in fluctuating populations may decrease due to repeated bottlenecks, but high levels of diversity are reported frequently (Berthier et al., 2005; Ehrich et al., 2009; García‐Navas et al., 2015; Gauffre et al., 2014; Ishibashi & Takahashi, 2021). Genetic variation is retained in fluctuating populations via several mechanisms, such as differences in dispersal patterns during fluctuations, crash phases with a short duration accompanied by weak genetic drift, and rapid accumulation of new alleles by mutation or immigration during a phase of increasing density (Gauffre et al., 2014; Norén & Angerbjörn, 2014). Because mutation is a rare event, individual dispersal (or migration) must be essential for maintaining genetic diversity in fluctuating arvicoline populations. When habitats are fragmented and dispersal is severely constrained in the surrounding habitat, genetic variation could be lost from each fragmented habitat during density fluctuations, and genetic diversity could gradually decrease in the whole population over time.

**FIGURE 1:**
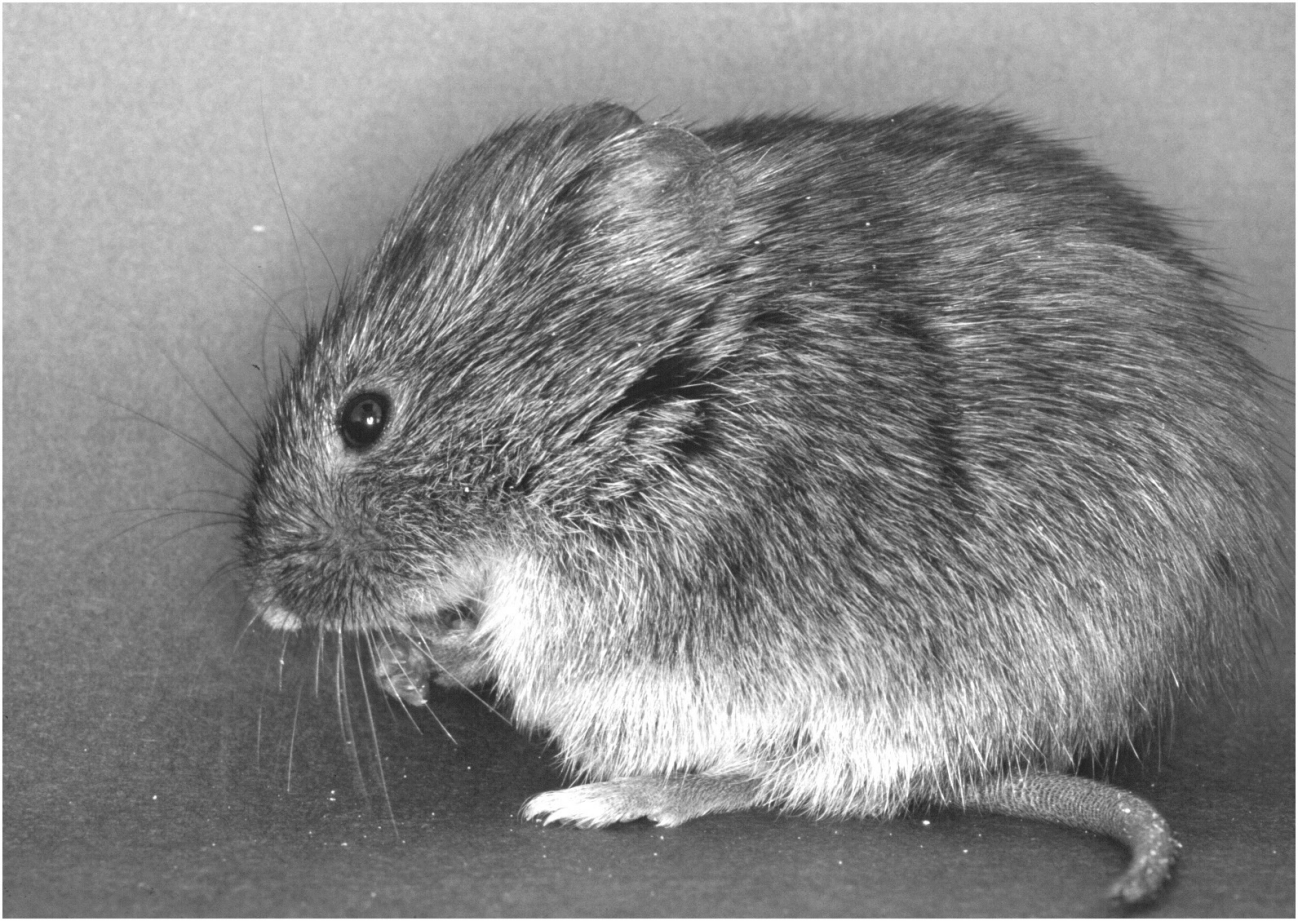
The gray‐sided vole, *Myodes rufocanus*. In Japan, the vole occurs only on Hokkaido, the northernmost island.

In Japan, the gray‐sided vole *Myodes rufocanus* occurs only on Hokkaido, the northernmost island (Figure 1). Most vole populations fluctuate in density within and across years (Saitoh, 1987; Saitoh et al., 1998). Here, I investigated whether genetic diversity in the gray‐sided vole was well preserved within and among fragmented habitats in an intensive farming region. The study site harbors many windbreak forests along the borders of plowed lands, and these forests are frequently inhabited by voles. By examining seasonal changes in the genetic structure of vole samples collected from the same windbreak forests twice a year, I investigated whether gene flow via individual dispersal was restrained among fragmented habitats scattered within a 20 km scale. In a previous 5‐year study involving two fixed grids (~0.5 ha each), the mean number of alleles per microsatellite DNA locus (allelic diversity) varied with density at a local scale but appeared to be preserved at a regional scale because of the density‐dependent dispersal behavior of individuals (Ishibashi & Takahashi, 2021). This previous study was performed on a peninsula, most of which was vegetated, and the study populations did not appear to be fragmented.

The gray‐sided vole is a small (~50 g) herbivorous rodent with a short life span (mean, 193 days for bred females; Saitoh, 1990). The vole has a male‐biased dispersal pattern; males are expected to disperse farther than females. Although voles do not disperse as much during the non‐breeding winter season (Ishibashi et al., 1998), during the breeding season individuals travel longer distances around sexual maturity (Saitoh, 1995), and mating competition and success may be linked to the dispersal of males (Kawata, 1989). During the breeding season, home‐range lengths were 16.3 ± 6.0 m (mean ± *SD*; range 10–40 m, *N* = 100) and 32.7 ± 12.1 (10–65 m, *N* = 64) for bred females and males, respectively (Ishibashi & Saitoh, 2008a). Within a 3 ha enclosure, females and males that bred in the year of birth dispersed and bred 19.3 m (median; interquartile range 10.1–42.1 m, *N* = 76) and 103.5 m (56.9–135.0 m, *N* = 46), respectively, from the estimated birthplace (Ishibashi & Saitoh, 2008a). Gray‐sided voles can disperse across cultivated fields (Dewa, 2002), but it is unknown how frequently they disperse across these habitats.

In the present study, I examined the temporal and spatial genetic structures of the study population using two DNA markers with different inheritance systems: that is, maternally inherited mitochondrial DNA (mtDNA) and biparentally inherited microsatellite DNA. First, the genetic structures of mtDNA were compared between sexes to clarify sexual differences in dispersal patterns. Second, to evaluate whether individual dispersal works well in preserving neutral nuclear genetic variations, genetic structure at microsatellite DNA loci was compared between seasons. Subsequently, genetic diversity was compared with data from a previous study performed in much less fragmented populations. In addition, cluster admixture proportions were explored using Bayesian clustering analysis. By comparing cluster admixture proportions of individuals between seasons and between sexes, I estimated how genetic admixture progressed within and among windbreak forests in association with individual dispersal. To my knowledge, it is not yet known how admixture of different clusters progresses by individual dispersal at the population level during density fluctuations. Clustering analysis has been used to evaluate the spatial genetic structure and admixture of lineages (Beichman et al., 2023; Dai et al., 2022; Flucher et al., 2021; Krojerová‐Prokešová et al., 2015). Only a few studies have revealed temporal changes in cluster admixture proportions. Day et al. (2022) investigated genetic diversity and structure in reintroduced American martens, *Martes americana*, in Wisconsin, which were sampled during two time periods separated by 10 years. Because migration among populations was apparent but the degree of admixture, estimated based on cluster admixture proportions, was low and declined over time, they inferred that martens successfully dispersed between populations but migrants did not contribute to genetic diversity, likely because of assortative mating (Day et al., 2022). In the aforementioned study of gray‐sided voles using two fixed grids, admixture proportions changed differently between the grids (Ishibashi & Takahashi, 2021). In one of the grids, into and from which individuals could migrate freely, the admixture proportions of three clusters changed before and after population crash events. After a crash, admixture proportions varied with time. In contrast, at the other grid, which was located in a partially dispersal‐limited space, the admixture proportions of two clusters changed markedly before and after crash events and were nearly fixed in a single cluster during density‐increase phases. Because the study grids were located far apart from one another, it is unknown how genetic admixture progresses among different sites in close proximity.

## 2 MATERIALS AND METHODS

### 2.1 Study site

**FIGURE 2:**
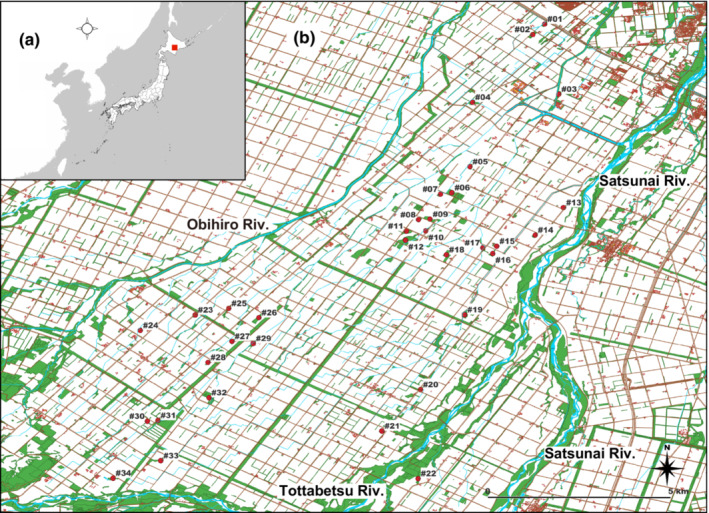
Study site and trapping sites. (a) Location of the study site in Hokkaido, Japan. (b) Location of trapping sites (red dots; #01–34; see Appendix S1 for precise locations). Most white areas are farmland. Green areas are bushes of *Sasa nipponica*, the main habitat of the gray‐sided vole, and riparian and windbreak forests, where *S*. *nipponica* may be distributed as understory vegetation. Brown lines in grid patterns and blue thin lines depict roads and runnels, respectively, the slopes of which were typically covered with vegetation, frequently including *S*. *nipponica*. The distribution of forests during the study period was obtained from Basic Map Information (Geospatial Information Authority of Japan), Hokkaido Government Opendata CC‐BY4.0, and the results of the 2011 Obihiro City Biodiversity Conservation Project (Obihiro City Government), which was modified with reference to a Landsat image (May 2006) in Google Earth Pro, as several of the windbreak forests have since disappeared.

Tissue samples were collected in the middle of the Tokachi Plain in Hokkaido, Japan. The plain is the largest farming region in Japan, where crops—such as beans, sugar beets, potatoes, and wheat—are rotated (Hokkaido Government, 2014). Settlement in this plain began at the end of the 19th century. Improvements to drainage were necessary to protect agriculture from cold and water damage, as wet soils cover about half of the total arable land area (Hokkaido Government, 2014). Productivity increased greatly after land improvement projects began in the 1960s, and the area was transformed into a representative cold‐hardiness field crop and dairy farming areas of Japan in the 1970s (Hokkaido Government, 2014). Many windbreak forests are located along the borders of plowed lands in the plain. Voles were trapped in 34 windbreak forests from northeast to southwest in an area between the Satsunai and Obihiro Rivers (Figure 2 and Appendix S1). Distances between trapping sites ranged from 0.35 to 20 km. Windbreak forests in the plain mainly (>80%) consisted of Japanese larch, *Larix kaempferi* (Nakagawa, 2018). Dwarf bamboo, *Sasa nipponica*, the main habitat of the gray‐sided vole, is distributed as understory vegetation. The study area has many low‐volume narrow roads in a grid pattern, as well as runnels (Figure 2). Because the slopes of these roads and runnels are covered with vegetation, they may facilitate vole movement.

### 2.2 Sampling tissues

With the permission of the Hokkaido Government, vole samples were collected from June 5 to July 29 (late spring–mid‐summer; hereafter referred to as spring) and September 5 to October 20 (fall) in 2007, without sacrificing individuals. The trapping sessions were conducted at the same position in all windbreak forests. During a trapping session, 30 Sherman‐type live traps were set in a 3 × 10 grid pattern at an interval of 10 m (~0.3 ha) with a handful of oats in each trap for two nights; that is, a total of 60 trap nights per session. Upon capture, three toes (one per foot) of each vole were clipped, sex was determined, and the vole was released. Clipped toes were stored in plastic bags at −10°C during a trapping session and at −80°C in the laboratory until DNA extraction.

### 2.3 DNA typing

Genomic DNA was extracted from samples (two toes per individual) using the conventional phenol‐chloroform method (Sambrook et al., 1989). The partial nucleotide sequences of the mtDNA control region were determined as described previously (de Guia et al., 2007), with some modifications. The most variable part (425 bp) was sequenced using primarily the internal primer HIP8, but also the primer Lpro, on an ABI PRISM 310 Genetic Analyzer (Applied Biosystems) with the software Sequencing Analysis 3.4.1. In addition, for 18 trapping sites with ≥17 samples, six microsatellite DNA loci (MSCRB01, MSCRB04, MSCRB07, MSCRB09, MSCRB11, and MSCRB13) were genotyped on an ABI PRISM 310 Genetic Analyzer with GeneScan 3.1.2 and Genotyper 2.5, as reported previously (Ishibashi & Takahashi, 2021). Furthermore, the sex of juveniles classified as sex‐unknown in the field, based on the external genitalia, was determined by the presence/absence of PCR products from two Y chromosome‐specific microsatellite DNA loci (MSCRY1 and MSCRY2; GenBank/DDBJ nos. AB300923 and AB300924). Two primer sets were used for PCR amplification: Y1F, 5′‐TGGCCAGCGTTTAGCCATTTCAC‐3′ and Y1R, 5′‐TGTCCTGTGCCATTTGTAAATC‐3′ for MSCRY1, and Y2F; 5′‐CTTACTGCTCTTACACCTAGT‐3′ and Y2R, 5′‐TGGTGGATGAGTTATGTGGTA‐3′ for MSCRY2. The amplification conditions for both loci were as follows: 95°C for 10 min, followed by 34 cycles of 20 s at 93°C, 15 s at 54°C, and 20 s at 72°C.

### 2.4 Analysis of mtDNA haplotypes

To identify factors related to the number of mtDNA haplotypes in each sex at trapping sites, generalized linear model (GLM) selection was performed using the glm function (family = Poisson) in R software (version 4.0.4; R Core Team, 2021). Through backward elimination (using the step function) to minimize the Akaike information criterion (AIC) value, the model with the smallest AIC value was selected as the best model (Burnham & Anderson, 2002). Four explanatory variables were considered: number of males, number of females, season (spring or fall), and area of windbreak forest in which the focal trapping site was located (Appendix S1). During model fitting, unusual data, which had a negative influence on the model fit, were detected using the residualPlots and influenceIndexPlot functions, implemented in the package car (Fox & Weiisberg, 2011), and excluded from the analysis. Collinear relationships among variables, that is, collinearity, were checked using the vif function in the car package. Overdispersion of data was tested with the dispersion test function in the package AER (Kleiber & Zeileis, 2008).

To assess the effect of male‐biased dispersal on spatial genetic structure, mtDNA haplotype frequencies were analyzed in each sex separately. Linearized *F*_ST_ (= *F*_ST_/(1–*F*_ST_); Slatkin, 1995), hereafter referred to as lin*F*_ST_, was calculated for all pairs of trapping sites with five or more samples with GenAlEx 6.51b2 (Peakall & Smouse, 2006, 2012). Mantel tests were performed with GenAlEx to assess the significance of correlations between geographic distance and lin*F*_ST_ in each season.

### 2.5 Analysis of microsatellite DNA genotypes

Using Micro‐Checker 2.2.3 (Van Oosterhout et al., 2004), the presence of null alleles at the microsatellite DNA loci was examined at each trapping site; spring and fall samples were analyzed separately. Hardy–Weinberg tests were performed online (https://genepop.curtin.edu.au/) using GENEPOP 4.7.5 (Raymond & Rousset, 1995). *F*_IS_, an index of the inbreeding of individuals relative to their population fragments (Frankham et al., 2017), was estimated with GenAlEx. Genetic diversity at each trapping site was estimated based on individual heterozygosity and the mean number of alleles per locus (allelic diversity). Individual heterozygosity was calculated from genotype data as follows: if an individual was homozygous at a locus, it was scored as 0, while if it was heterozygous, it was scored as 1; the values were averaged over all loci (Coulson et al., 1998). Allelic diversity was estimated with GenAlEx.

To identify factors related to genetic diversity, linear regression model (LM) selection was performed for the mean individual heterozygosity and mean number of different alleles at trapping sites (response variables) using the lm function in R. The best model was identified as the GLM analysis mentioned previously. As predictors, the number of individuals, seasons, and area of windbreak forests were considered in the LM analysis.

To compare spatial genetic structure between seasons, lin*F*_ST_ values were calculated for all pairs of trapping sites with five or more samples using GenAlEx. Because the sample size at each trapping site was not very large, males and females were not separated in the analysis. Mantel tests were performed with GenAlEx to examine correlations between geographic distance and lin*F*_ST_ in each season.

### 2.6 STRUCTURE analysis

To examine how genetic admixture progressed within and among windbreak forests, Bayesian clustering analysis was performed using STRUCTURE 2.3.4 (Falush et al., 2003; Hubisz et al., 2009; Pritchard et al., 2000), as described previously (Ishibashi & Takahashi, 2021), with some modifications. The admixture proportions of clusters within individuals were estimated using the admixture model. The number of clusters (*K*) considered varied from 1 to 10. Each run, replicated 100 times, comprised a burn‐in period of 10^4^ and 10^4^ iterations under default settings except option LOCPRIOR, for which trapping sites of individuals were used as prior information. The most likely number of clusters was inferred based on ad hoc Δ*K* statistics (Evanno et al., 2005).

### 2.7 Other statistical analyses

Statistical analyses not specified above were performed using R software.

## 3 RESULTS

### 3.1 Samples

DNA samples were collected from 673 voles at 34 trapping sites (161 males and 169 females in spring; 155 males and 188 females in fall). The density at trapping sites ranged from low to medium (0–38.3 per 0.5 ha equivalent). Based on the reproductive activity of gray‐sided voles in Hokkaido, Japan (Ishibashi & Saitoh, 2008b), the spring samples appeared to consist of overwintered adults born in the fall of 2006 and their offspring born in 2007, while the fall samples consisted of adults born in 2007 and their offspring.

### 3.2 mtDNA diversity

**FIGURE 3:**
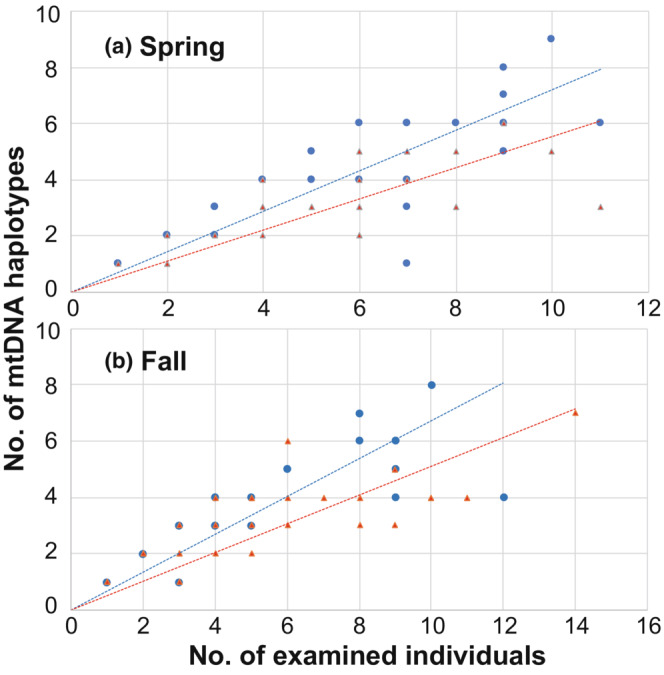
Relationship between the number of examined individuals and the number of different mitochondrial DNA haplotypes at trapping sites. Blue circles and red triangles indicate males and females, respectively. Regression lines: males in spring, *y* = 0.7214 × *x* (*R* ^2^ = .925); females in spring, *y* = 0.5555 × *x* (*R* ^2^ = .908); males in fall, *y* = 0.6709 × *x* (*R* ^2^ = .918); females in fall, *y* = 0.5106 × *x* (*R* ^2^ = .901).

The sequence of part of the mtDNA control region (425 bp) was determined for 673 individuals. There were 41 variable sites among the sequences, defining 76 haplotypes (Appendix S2). These haplotypes differed from each other by 0 to 11 sites; because of this small amount of differentiation, their phylogenetic relationships were ambiguous (Appendix S3). Although a total of 62 haplotypes were counted in each season, 1–11 haplotypes and 1–9 haplotypes were observed at each trapping site in spring and fall, respectively (spring, mean ± *SD* = 5.1 ± 2.67, *N* = 33; fall, 5.2 ± 2.28, *N* = 34; Appendix S4). In each season, the number of mtDNA haplotypes at trapping sites was significantly positively correlated with the number of examined individuals in both sexes (Spearman's rank correlation test; males in spring, *ρ* = .857, *p* < .0001; females in spring, *ρ* = .772, *p* < .0001; males in fall, *ρ* = .900, *p* < .0001; females in fall, *ρ* = .804, *p* < .0001; Figure 3). The best models in the GLM analysis for the number of mtDNA haplotypes indicated that the number of males captured at trapping sites was the only explanatory variable related to the number of haplotypes detected in males, whereas the number of females was the only explanatory variable related to the number of haplotypes in females (Tables 1 and 2, respectively). The number of different‐sex individuals, season, and windbreak forest area were not closely related to the number of haplotypes in either sex. The variance inflation factors (VIFs) for the explanatory variables were <1.65, which suggested slight collinearity (Fox & Weiisberg, 2011). Overdispersion was not found in the dataset (males, *z* = −15.969, *p* = 1; females, *z* = −14.729, *p* = 1).

**TABLE 1:** Generalized linear models of the number of mitochondrial DNA haplotypes in males at trapping sites constructed using the glm function in R (family = Poisson).

Models	AIC
1. Nm + Nf + Season + log(Area)	208.58
2. Nm + Season + log(Area)	206.59
3. Nm + log(Area)	204.67
4. Nm	202.81

*Note*: Four explanatory variables were considered: Nm, number of examined males; Nf, number of examined females; Season, spring or fall (as a factor); Area, area of windbreak forest (ha) in which the focal trapping site was located. Using the step function, the final (best) model (Model 4) was selected. Three cases (site #13 in spring, site #24 in spring, and site #24 in fall) were excluded from the analysis.

**TABLE 2:** Generalized linear models of the number of mitochondrial DNA haplotypes in females at trapping sites constructed using the glm function in R (family = Poisson).

Models	AIC
1. Nm + Nf + Season + log(Area)	213.14
2. Nm + Nf + Season	211.2
3. Nf + Season	209.46
4. Nf	207.66

*Note*: Four explanatory variables were considered: Nm, number of males; Nf, number of females; season, spring or fall (as a factor); and area, the area of windbreak forest (ha) in which the focal trapping site was located. Using the step function, the final (best) model (Model 4) was selected. One case (site #02 in fall) was excluded from the analysis.

### 3.3 Spatial structure of mtDNA haplotypes

**FIGURE 4:**
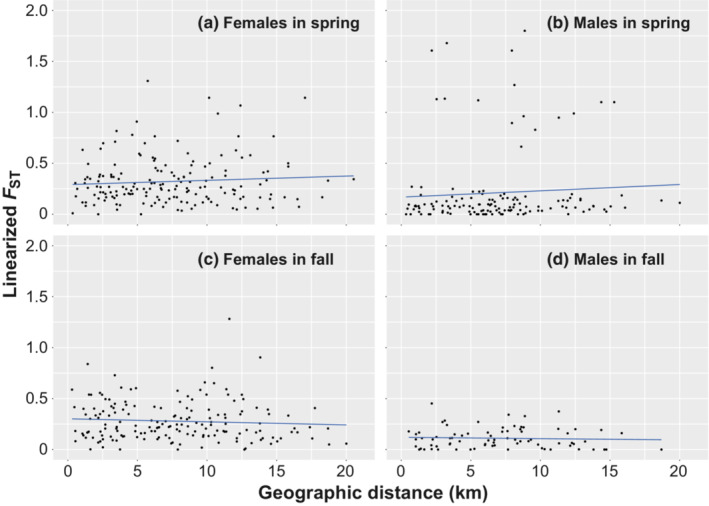
Spatial genetic structure as revealed by mitochondrial DNA haplotypes. No correlation was observed between the geographic distance and linearized *F*_ST_ in any combination of sex and season (Mantel test: (a) *R*_*xy*_ = 0.079, *p* = .227; (b) *R*_*xy*_ = 0.069, *p* = .296; (c) *R*_*xy*_ = −0.072, *p* = .243; (d) *R*_*xy*_ = −0.054, *p* = .392). In spring samples of males (b), linearized *F*_ST_ values >0.5 were observed in all combinations between trapping site #24 and the other 16 trapping sites. Regression lines show trends.

Lin*F*_ST_ values were calculated among trapping sites with five or more samples for each sex (males, 17 sites in spring and 13 sites in fall; females, 19 sites in both seasons). In both sexes, no correlation was observed between lin*F*_ST_ and geographic distance between trapping sites in either season (Mantel test, *p* > .2 for all; Figure 4). On average, lin*F*_ST_ values in females were greater than in males in both seasons (spring, mean ± *SD* = 0.32 ± 0.234 [*N* = 171] in females and 0.21 ± 0.376 [*N* = 136] in males; fall, 0.28 ± 0.196 [*N* = 171] in females and 0.11 ± 0.100 [*N* = 78] in males). In females, although variance in spring was significantly greater than in fall (*F* test, *F* = 1.422, *df* = 170 and 170, *p* = .011, one‐tailed), mean values were not different between seasons (Wilcoxon rank sum test, *W* = 13,192, *p* = .118). In males, spring samples showed marked variability because lin*F*_ST_ values >0.5 were observed in all combinations between trapping site #24 and the other 16 sites (Figure 4b), because a single haplotype was detected at site #24 in spring. Excluding the site #24 samples, the mean and variance (spring, mean ± *SD =* 0.08 ± 0.067, *N* = 120; fall, 0.09 ± 0.078, *N* = 66) were similar between seasons in males (mean, *W* = 4154, *p* = .581; variance, *F* = 0.724, *df* = 119 and 65, *p* = .129). In addition, the mean and variance in males were significantly smaller than in females in both seasons (*p* < .0001 for all tests; one‐sided).

### 3.4 Microsatellite DNA diversity

Genotypes at six microsatellite DNA loci were determined for 494 voles (245 in spring and 249 in fall) from 18 trapping sites where 17 or more voles were sampled. In total, 16–24 different alleles were detected at those loci (Appendix S5). Hardy–Weinberg exact tests showed that the observed genotypes at each site conformed to expectations regarding random union of gametes at all loci in both seasons (Bonferroni‐corrected *p* > .05/6 for all). *F*_IS_ values did not differ from zero (spring, *F*_IS_ = −0.077, *p* = 1; fall *F*_IS_ = −0.038, *p* = 1), suggesting that no inbreeding occurred in windbreak forests. Linkage disequilibrium was not found among the loci (Bonferroni corrected *p* > .05/15 for all). Micro‐Checker analysis suggested the presence of null alleles at three loci at three trapping sites (one in spring and two in fall) because of homozygote excess (Appendix S6). At each of the trapping sites, the presence of null alleles was not suggested at another trapping time. Null alleles are likely to be very rare in the population, or homozygote excess occurred by chance because of the small sample size. All genotype data were included in subsequent analyses without excluding any loci.

**FIGURE 5:**
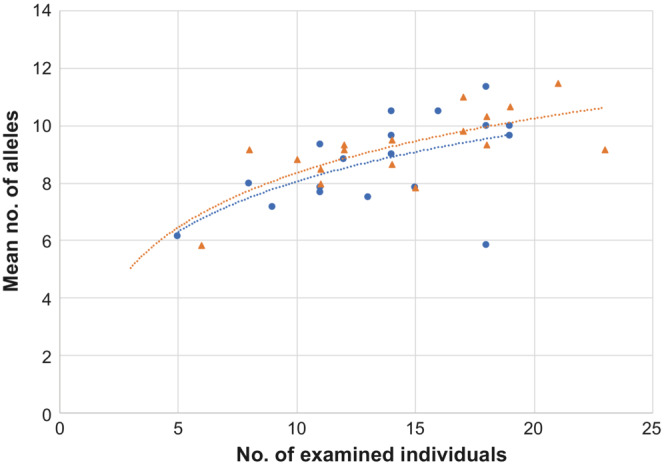
Relationship between the number of examined individuals and the mean number of different microsatellite DNA alleles at trapping sites. Blue circles and red triangles denote spring and fall samples, respectively. Regression lines: spring, *y* = 2.53 × ln(*x*) + 2.2351 (*R* ^2^ = .324); fall, *y* = 2.737 × ln(*x*) + 2.0517 (*R* ^2^ = .537).

The mean number of observed alleles was significantly positively correlated with the number of examined individuals in both seasons (Spearman's rank correlation test: spring, *ρ* = .557, *p* = .016; fall, *ρ* = .670, *p* = .003). The relationships between the number of individuals and the mean number of alleles were similar between seasons (Figure 5).

Individual heterozygosity was high at almost all trapping sites in both seasons and no difference was observed between seasons; the mean ± *SE* for all individuals was 0.88 ± 0.009 in spring and 0.87 ± 0.009 in fall (Wilcoxon signed rank test, *V* = 94, *p* = .420; Table 3). Because LM analysis indicated that the best model comprised only the intercept term, mean heterozygosity at trapping sites was not explained by any of the variables considered (Table 4). No collinearity was detected in the analysis (VIFs < 1.04 for all variables).

**TABLE 3:** Genetic diversity at six microsatellite DNA loci at two trapping times.

Trapping site no.	Spring					Fall				
	*N*	Individual heterozygosity		No. of alleles per locus		*N*	Individual heterozygosity		No. of alleles per locus	
		Mean	*SE*	Mean	*SE*		Mean	*SE*	Mean	*SE*
02	11	0.89	0.034	9.3	0.56	10	0.83	0.035	8.8	0.79
03	14	0.88	0.027	10.5	0.34	21	0.86	0.033	11.5	0.67
09	11	0.86	0.038	7.7	0.71	6	0.72	0.111	5.8	0.40
10	18	0.89	0.030	10.0	0.68	18	0.91	0.020	9.3	0.67
11	19	0.85	0.036	9.7	0.49	11	0.82	0.042	8.0	0.63
12	18	0.88	0.035	11.3	0.71	3^a^	–	–	–	–
13	19	0.84	0.041	10.0	0.58	15	0.83	0.043	7.8	0.70
14	16	0.89	0.033	10.5	0.72	14	0.92	0.029	9.5	0.34
18	15	0.93	0.027	7.8	0.60	12	0.85	0.032	9.3	0.76
19	11	0.88	0.032	7.8	0.48	19	0.93	0.029	10.7	0.71
23	5	0.90	0.041	6.2	0.48	17	0.87	0.034	11.0	0.52
24	18	0.82	0.032	5.8	0.54	23	0.88	0.028	9.2	0.91
25	12	0.88	0.036	8.8	0.70	8	0.90	0.070	9.2	0.70
27	8	0.94	0.030	8.0	0.77	11	0.92	0.035	8.5	0.22
28	14	0.83	0.035	9.0	0.52	14	0.89	0.041	8.7	0.42
30	9	0.87	0.046	7.2	0.54	12	0.94	0.024	9.2	0.60
32	14	0.92	0.042	9.7	0.92	18	0.84	0.034	10.3	0.49
34	13	0.91	0.045	7.5	0.62	17	0.84	0.039	9.8	0.48
Grand^b^	–	0.88	0.009	8.7	0.20	–	0.87	0.009	9.2	0.19

*Note*: *N*, number of individuals examined.

^a^ Data excluded from the calculation because of a small sample size.

^b^ Grand mean and *SE* over sites and loci.

**TABLE 4:** Linear regression models of the mean individual heterozygosity at trapping sites constructed using the lm function in R.

Models	AIC
1. NoInd + Season + log(Area)	−222.26
2. NoInd + log(Area)	−224.23
3. NoInd	−226.18
4. 1 (intercept term only)	−226.60

*Note*: Three explanatory variables were considered: NoInd, number of examined individuals; season, spring or fall; and area, area of windbreak forest (ha) in which the focal trapping site was located. Using the step function, the final (best) model (Model 4) was selected, which comprised only the intercept term. One case (site #24 in fall) was excluded from the analysis.

The mean number of alleles per trapping site ranged from 5.8 to 11.5 (Table 3). The best LM model with respect to the mean allele number included the number of examined individuals as a predictor (Table 5). No collinearity was detected in the analysis (VIFs < 1.01 for all variables).

**TABLE 5:** Linear regression models of the mean number of alleles per locus at trapping sites constructed using the lm function in R.

Models	AIC
1. NoInd + Season + log(Area)	−5.60
2. NoInd + log(Area)	−7.13
3. NoInd	−8.38

*Note*: Three explanatory variables were considered: NoInd, number of examined individuals; season, spring or fall; and area, area of windbreak forest (ha) in which the focal trapping site was located. Using the step function, the final (best) model (Model 3) was selected. Two cases (site #24 in spring and site #24 in fall) were excluded from the analysis.

### 3.5 Seasonal changes in the spatial genetic structure of microsatellite DNA

**FIGURE 6:**
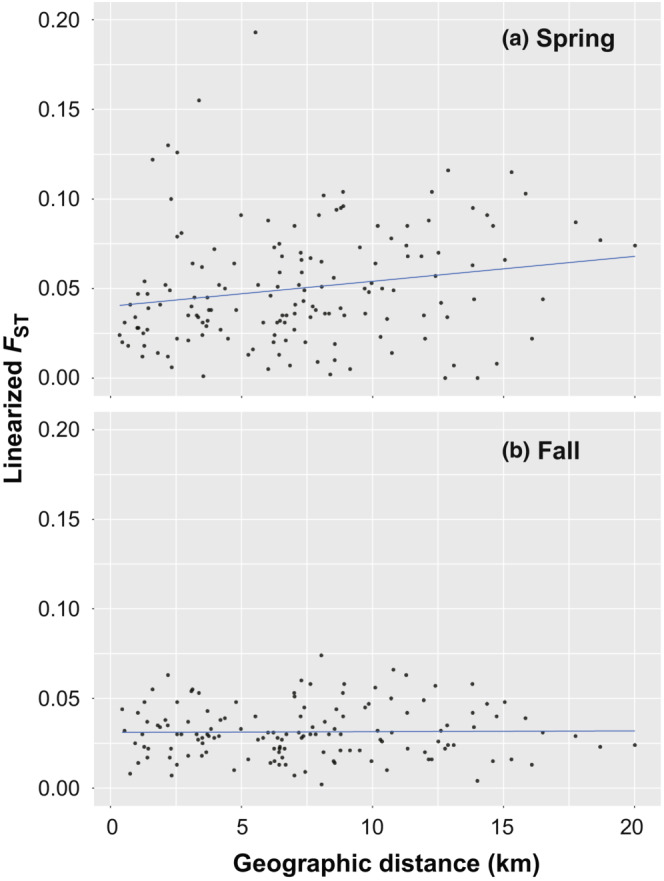
Spatial genetic structure as revealed by microsatellite DNA (a) Spring: Mantel test, *R*_*xy*_ = 0.188, *p* = .094. (b) Fall: Mantel test, *R*_*xy*_ = 0.014, *p* = .480. Regression lines show trends.

Lin*F*_ST_ values were calculated among trapping sites with five or more samples (18 sites in spring and 17 sites in fall). The spatial genetic structure of microsatellite DNA loci decreased considerably from spring to fall (Figure 6). The mean and variance of lin*F*_ST_ values (spring, mean ± *SD* = 0.05 ± 0.033, *N* = 153; fall, 0.03 ± 0.015, *N* = 136) were significantly smaller in fall (Wilcoxon rank‐sum test for means: *W* = 14,124, *p* < .0001; *F* test for variances, *F* = 4.992, *df* = 152 and 135, *p* < .0001). No significant correlation was detected between geographic distance and lin*F*_ST_ in either season (Mantel test: spring, *R*_xy_ = 0.188, *p* = .094; fall, *R*_xy_ = 0.014, *p* = .480), although the significance was marginal in spring.

### 3.6 Seasonal changes in cluster admixture proportions

**FIGURE 7:**
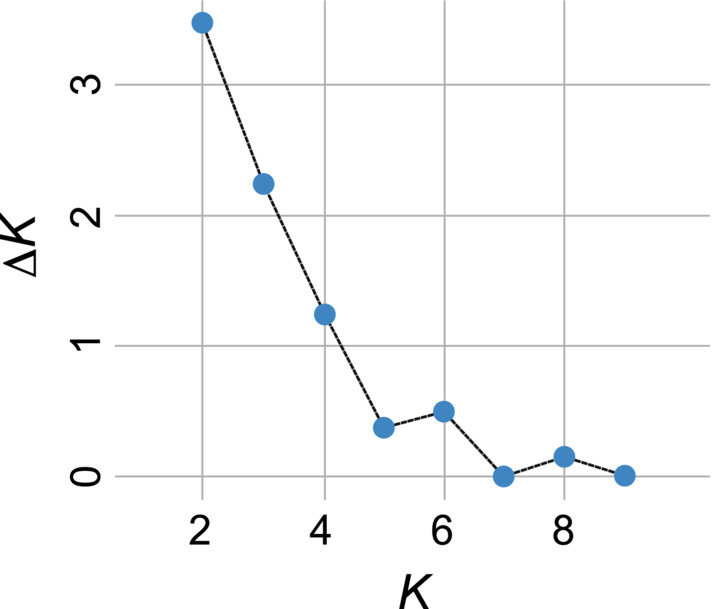
Plot of Δ*K* statistics.

**FIGURE 8:**
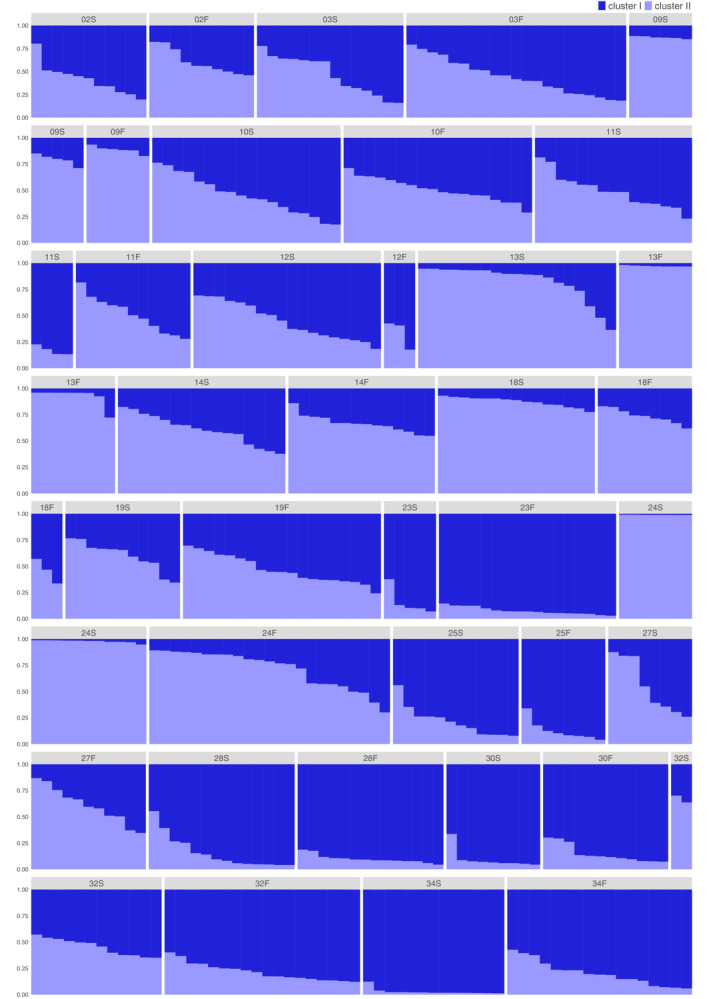
Cluster admixture proportions for 494 individuals at 18 trapping sites in two seasons at *K* = 2. Each individual is represented by a vertical bar and admixture proportions are denoted by different colors; clusters I and II are indicated by dark and pale blue, respectively. Numbers and letters indicate trapping sites and sampling seasons, respectively (S, spring; F, fall). For example, 24S indicates samples from site #24 in the spring.

**FIGURE 9:**
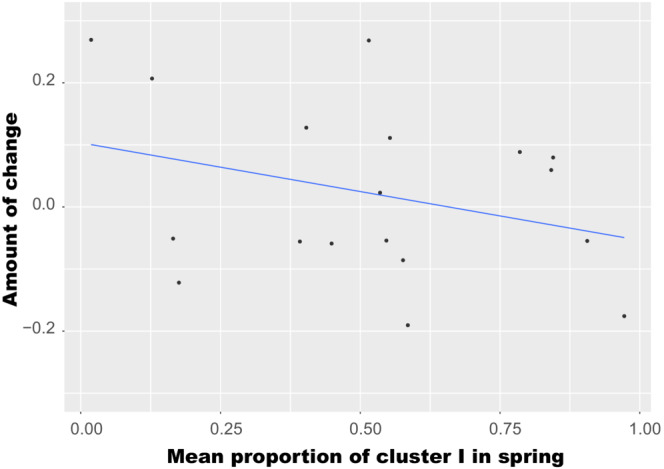
Negative relationship between the mean admixture proportion of cluster I in spring and the degree of change in admixture proportions between spring and fall at *K* = 2. The correlation was marginally significant (Spearman's rank‐correlation test, *S* = 1300, *p* = .083). The regression line presents the trendline.

**FIGURE 10:**
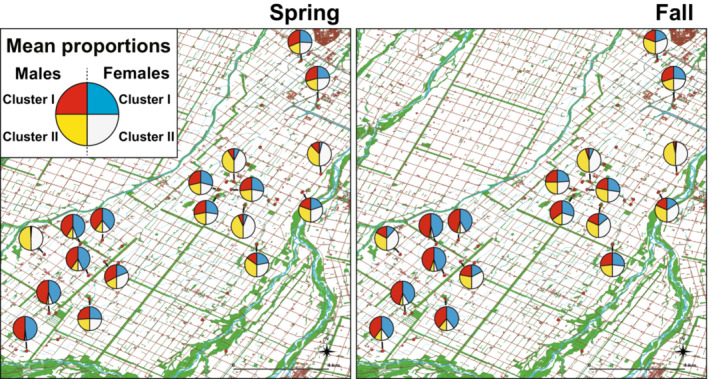
Mean admixture proportions of clusters for each sex at *K* = 2.

**FIGURE 11:**
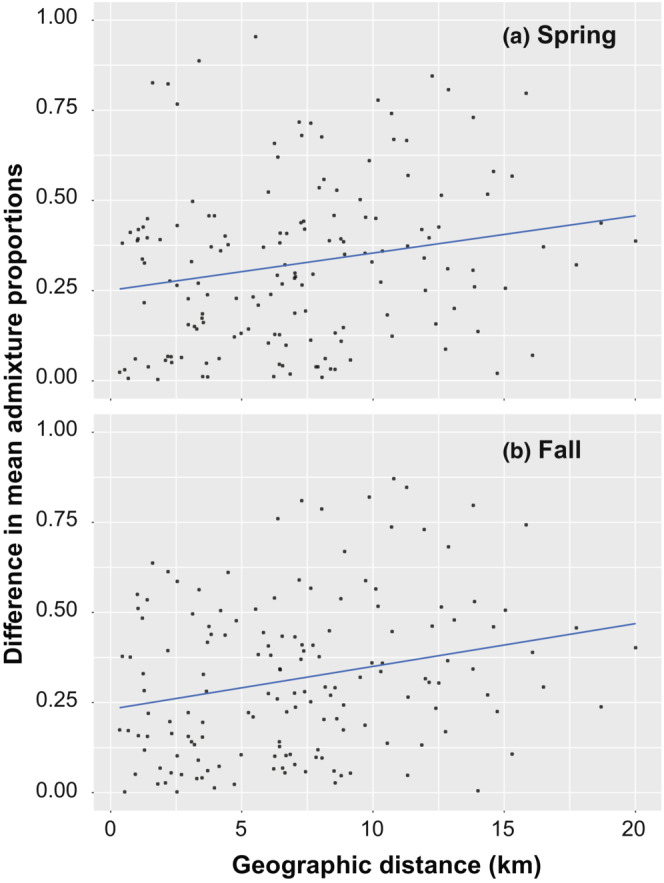
Positive relationship between the geographic distance between trapping sites and differences in mean admixture proportions of cluster I at *K* = 2. (a) Spring: Mantel test, *R*_*xy*_ = 0.200, *p* = .046. (b) Fall: Mantel test, *R*_*xy*_ = 0.242, *p* = .013. Regression lines present trendlines.

In the STRUCTURE analysis, the most probable number of clusters (*K*) was 2 for 494 individuals from 18 trapping sites, based on Δ*K* statistics (Figure 7). The 18 trapping sites had varying proportions of clusters I and II within individuals in both seasons at *K* = 2 (Figure 8). Some trapping sites were nearly occupied by one of the clusters in both seasons (#24 and #34 in spring; #13 and #23 in fall). At a second probable cluster number of 3 (*K* = 3), the trapping sites also had varying proportions of three clusters within individuals (Appendix S7). The mean admixture proportions of a cluster increased or decreased at each trapping site between seasons. At *K* = 2, the average mean admixture proportion of cluster I among trapping sites was 0.522 ± 0.279 (mean ± *SD*) for spring samples and 0.543 ± 0.270 for fall samples, and the difference between seasons was not significant (Wilcoxon signed‐rank exact test: *V* = 70, *p* = .523). However, a weak negative correlation was observed between the mean admixture proportion of cluster I in spring and change in mean admixture proportion of cluster I between seasons, although the significance was marginal (Spearman's rank‐correlation test, *p* = .083; Figure 9). The admixture proportions of clusters were spatially heterogeneous (Figure 10). A significant positive correlation was observed between the geographic distance between trapping sites and the difference in the mean admixture proportions in both seasons (Mantel test, *p* = .046 in spring, *p* = .013 in fall; Figure 11).

When the admixture proportions were analyzed for each sex separately, no significant difference was detected between seasons in the mean admixture proportion of cluster I or its variance in either sex (Wilcoxon signed‐rank test for means: females, *V* = 76, *p* = .702; males, *V* = 73, *p* = .610; *F* test for variances: females, *F* = 1.139, *df* = 17 and 17, *p* = .792; males, *F* = 1.040, *df* = 17 and 17, *p* = .937; Table 6). At each trapping site, females and males were consistent in terms of the direction of change in the mean admixture proportions of cluster I between seasons (Table 6 and Figure 10). However, the absolute change in males was greater than in females (Wilcoxon signed‐rank test, *V* = 22, *p* = .002, one‐tailed), and the average absolute value in males was 1.65‐fold greater than that in females (0.147 vs. 0.089).

**TABLE 6:** Mean admixture proportions of cluster I and degree of change between seasons at 18 trapping sites.

Trapping site no.	Females				Males			
	Spring	Fall	Amount of change^a^	Abs	Spring	Fall	Amount of change^a^	Abs
02	0.520	0.400	**−0.120**	0.120	0.622	0.387	**−0.235**	0.235
03	0.491	0.528	0.038	0.038	0.580	0.591	0.011	0.011
09	0.150	0.131	**−0.019**	0.019	0.174	0.098	**−0.076**	0.076
10	0.557	0.549	**−0.009**	0.009	0.538	0.436	**−0.102**	0.102
11	0.582	0.483	**−0.099**	0.099	0.570	0.497	**−0.073**	0.073
12	0.549	0.595	0.045	0.045	0.557	0.699	0.142	0.142
13	0.082	0.037	**−0.046**	0.046	0.244	0.065	**−0.178**	0.178
14	0.401	0.322	**−0.079**	0.079	0.385	0.362	**−0.023**	0.023
18	0.118	0.287	0.169	0.169	0.134	0.358	0.224	0.224
19	0.441	0.534	0.093	0.093	0.304	0.529	0.224	0.224
23	0.886	0.919	0.033	0.033	0.818	0.952	0.134	0.134
24	0.022	0.233	0.211	0.211	0.013	0.338	0.325	0.325
25	0.779	0.833	0.055	0.055	0.792	0.915	0.122	0.122
27	0.381	0.349	**−0.032**	0.032	0.652	0.439	**−0.213**	0.213
28	0.863	0.910	0.047	0.047	0.803	0.885	0.082	0.082
30	0.887	0.824	**−0.063**	0.063	0.945	0.906	**−0.039**	0.039
32	0.511	0.789	0.278	0.278	0.518	0.777	0.258	0.258
34	0.966	0.796	**−0.170**	0.170	0.978	0.797	**−0.181**	0.181
Mean	0.510	0.529	–	0.089	0.535	0.557	–	0.147
*SD*	0.290	0.272	–	0.074	0.280	0.275	–	0.089

Abbreviation: Abs, absolute degree of change.

^a^ Negative values are shown in bold.

## 4 DISCUSSION

Gray‐sided voles have been captured on a routine schedule to predict population outbreaks of the vole at several forests near the study site (17–31 km away). In these forests, relatively low peaks were observed in 2004–2005; a major decrease occurred in 2006; and the next peak was not observed until 2008 (Appendix S8). Given patterns of vole population fluctuations in the northern hemisphere (Andreassen et al., 2021), a population crash may have occurred during the winter of 2005–2006. At the study site, therefore, 2006 and 2007 (the study period) corresponded to the density‐increase phase between crash and peak phases because density fluctuations synchronize among close populations in this region (Saitoh et al., 1998). Frequent individual dispersal occurs soon after population crash events (Ishibashi & Takahashi, 2021). Individuals must have dispersed actively during the breeding season in 2006 and 2007 at the study site.

**FIGURE 12:**
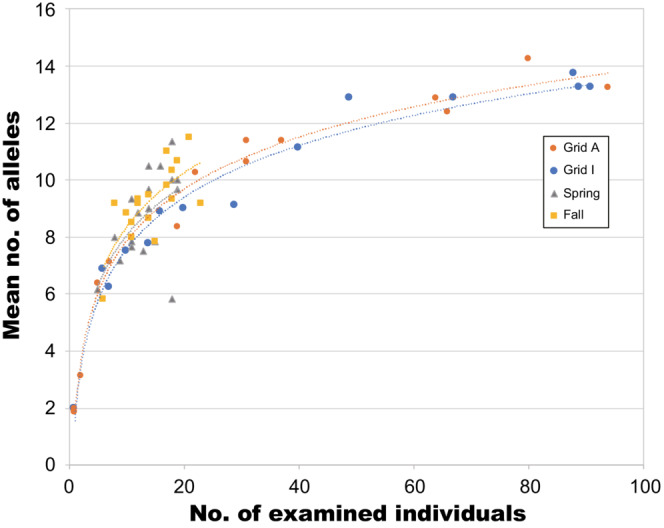
Relationship between the number of examined individuals and the mean number of different alleles (spring and fall) in this and a previous study (Grids A and I; Ishibashi & Takahashi, 2021). Regression lines: Grid A, *y* = 2.6268 × ln(*x*) + 1.8139; Grid I, *y* = 2.6154 × ln(*x*) + 1.5599.

In the present study, the number of mtDNA haplotypes at trapping sites was positively correlated with the number of examined individuals of each sex (Figure 3). Despite mtDNA being maternally inherited, the number of haplotypes in males was not closely related to the number of females but was instead related to the number of males (Table 1). In addition, the lin*F*_ST_ values among trapping sites were markedly lower in males than in females in both seasons (see Figure 4). The spring samples consisted of overwintered adults born in the fall of 2006 and their offspring born in 2007, while the fall samples consisted of adults born in 2007 and their offspring. Compared to females, males must have dispersed further during the breeding seasons in 2006 and 2007. At microsatellite DNA loci, individual heterozygosity was high at almost all trapping sites (Table 3) and was not closely related to the number of individuals, that is, density (Table 4). However, allelic diversity was closely related to density (Table 5), and the two variables were positively correlated in both seasons (Figure 5). Furthermore, genetic differentiation among trapping sites greatly decreased in fall (Figure 6). These findings indicate that in 2006 and 2007, gene flow driven by male‐biased dispersal generated a homogeneous spatial genetic structure at the study site. In a previous study, in which vole samples were collected at two fixed sites (0.5 ha each) in a heavily vegetated area of Hokkaido three times a year for 5 years, the number of alleles gradually plateaued during medium‐to‐high density periods (Ishibashi & Takahashi, 2021). Although the variation in mean values among trapping sites was large in the present study, the relationship between the number of examined individuals and the mean number of alleles was similar to the previous study (Figure 12). These results suggest that vole movement was not strongly inhibited in the cultivated fields, and allelic diversity at the present study site was preserved as well as in much less fragmented populations.

Although no significant relationship was observed between geographic distance and genetic differentiation among trapping sites for either DNA marker, a weak positive spatial correlation, the pattern of isolation by distance (IBD), was found in microsatellite DNA in spring (Figure 6a). Until the early spring of 2007, there may have been an IBD pattern at microsatellite DNA loci. Considerable differentiation may have arisen among windbreak forests after the population crash event in the winter of 2005–2006; subsequently, active individual dispersal may have temporarily generated an IBD pattern in 2006. Because of continuous gene flow, however, this effect may have almost disappeared by the time of sample collection in 2007.

In the STRUCTURE analysis, admixture proportions of clusters were spatially heterogeneous (Figure 10). Closer trapping sites showed more similar mean values of cluster admixture proportions (Figure 11). Just after the population crash event, the admixture proportions of clusters may have been most heterogeneously distributed at the study site in early 2006, and a positive correlation between distance and differences in the cluster proportions might not have been observed. Individual dispersal between neighboring windbreak forests likely explains the positive correlation between distance and differences in cluster admixture proportions.

At the trapping sites, the mean admixture proportions of a cluster increased or decreased between seasons. At *K* = 2, the mean admixture proportion of one cluster (and its variance) did not differ between seasons in either sex (Table 6). Females and males were consistent in terms of the directions of changes in the mean admixture proportions between seasons at each trapping site (Table 6). However, the degree of change in males was greater than in females at most sites (1.6 times on average). Considering the difference in genetic differentiation of mtDNA between sexes (Figure 4), many immigrated males and their offspring of both sexes may occur in the fall samples. At a given trapping site, therefore, the direction and degree of change in admixture proportions may be affected by their inclusion in the samples, although vole emigration from trapping sites also affects the direction and degree of change to some extent.

In a previous 5‐year study, after a population crash, admixture proportions varied with time and did not become fixed in a single cluster at one of the study grids, into and from which individuals could migrate freely (Ishibashi & Takahashi, 2021). In the present study, the mean admixture proportion of a cluster at trapping sites in spring was negatively correlated with the degree of change between seasons in its mean proportion, although its statistical significance was marginal (Figure 9). This finding suggests that individual dispersal promotes the admixture of two clusters around one half (0.5) at each trapping site. Admixture proportions of clusters changed considerably before and after the population crash events in the previous study (Ishibashi & Takahashi, 2021). Although vole samples around a crash event were not examined in the present study, if samples are examined around a crash event, more clusters might be detected in a STRUCTURE analysis. In this study, when the number of clusters was the second probable number of three, a negative correlation was also observed between the mean admixture proportion in spring and the degree of temporal change in mean admixture proportions (Appendix S9). Similarly, when more clusters are detected as a most probable cluster number *K* in a STRUCTURE analysis, mean admixture proportions of clusters would gradually approach 1/*K* and vary around it at each trapping site during density‐increase phases. Long‐term data involving population crash events is needed to verify the role of individual dispersal in the genetic admixture of fluctuating populations.

In the work by Ishibashi and Takahashi (2021), at one of the two study grids located in a partially dispersal‐limited space, the admixture proportions of two clusters were nearly fixed in a single cluster during density‐increase phases. In the present study, some trapping sites were nearly occupied by one of the clusters (Figure 8), although it is unknown whether the occupation observed in fall means the fixation of a cluster in the windbreak forests. In the Tokachi Plain, beans, sugar beets, potatoes, and wheat are grown as the primary crops; these are rotated, for example, in the order beans, potatoes, wheat, and sugar beets, as a cycle to avoid consecutive crop failures (Hokkaido Government, 2014). The wheat harvest season is in July; sugar beet and potato seedlings are planted in April; and beans are sown in May. After the wheat harvest until the following spring, the fields are frequently left bare for more than 8 months. During this period, many windbreak forests are completely or partially surrounded by bare fields without cover plants. This scenario may completely or partially limit vole migration routes into the windbreak forests during the summer–fall breeding season and the early‐spring breeding season, resulting in the occupation of a single cluster in the forests in the late spring (e.g., trapping sites #24 and #34). Furthermore, beans, which are harvested in late fall, may not provide appropriate cover for voles while the plants are growing. In this case, migration routes are likely to be limited during the spring–summer reproductive season, resulting in the occupation of a single cluster in fall (e.g., trapping sites #13 and #23). The STRUCTURE analysis results suggest that individual dispersal among windbreak forests depends on the conditions of surrounding fields. Further studies should analyze variation in cluster admixture proportions in relation to crop and field conditions surrounding windbreak forests.

Individual dispersal plays an important role in preserving genetic diversity in fluctuating populations of arvicoline rodents (Gauffre et al., 2014; Norén & Angerbjörn, 2014). In the present study, allelic diversity was preserved as well as in a previous study of much less fragmented populations (Ishibashi & Takahashi, 2021). Although individual dispersal may depend on the condition of surrounding fields, gene flow did not appear to be very restrained among isolated windbreak forests scattered within the intensive farming region. Moreover, male‐biased dispersal led to mixed genetic variation among fragmented windbreak forests, promoting genetic homogeneity in the study population. Therefore, genetic admixture by male‐biased dispersal must be crucial for the preservation of neutral nuclear genetic variation in fluctuating populations of the gray‐sided vole. Global warming and increased occurrence of extreme weather events caused by climate change may affect patterns of population fluctuations in small rodents (Andreassen et al., 2021). The present study was performed well before current rates of change; thus, the recent climate may currently be causing changes in the fluctuation cycle (see Appendix S8), thereby affecting the dispersal pattern of gray‐sided voles. Future studies should compare the genetic diversity observed herein to current and future populations at the same study site.

## AUTHOR CONTRIBUTIONS

**Yasuyuki Ishibashi:** Conceptualization (equal); data curation (equal); formal analysis (equal); funding acquisition (equal); investigation (equal); methodology (equal); project administration (equal); resources (equal); software (equal); supervision (equal); validation (equal); visualization (equal); writing – original draft (equal); writing – review and editing (equal).

## CONFLICT OF INTEREST STATEMENT

None declared.

## Supporting information


Appendix S1–S9.

## Data Availability

mtDNA sequences: GenBank/DDBJ accession nos. LC745586–LC745661. Genetic data on microsatellite DNA and mtDNA at trapping sites: Dryad doi: 10.5061/dryad.gmsbcc2sm.
