# Supplementary material for: Preservation of genetic diversity in a highly fragmented population of the gray‐sided vole Myodes rufocanus in an intensive farming region
Source: Ecol Evol. 2023 Sep 19;13(9):e10472. doi: 10.1002/ece3.10472 (PMC10509600; doi:10.1002/ece3.10472)

Appendices

Appendix S1. Locations of the trapping sites and the area of the windbreak forests in which trapping was conducted.

Trapping site #	Latitude (º)	Longitude (º)	Area (ha)
01	42.866586	143.141722	2.52
02	42.863000	143.137444	2.44
03	42.843072	143.146333	1.44
04	42.840547	143.117667	3.20
05	42.819158	143.116889	0.88
06	42.810417	143.110833	5.56
07	42.809844	143.106944	2.92
08	42.801308	143.099556	1.56
09	42.801511	143.102833	3.16
10	42.797508	143.102111	0.52
11	42.797386	143.095500	3.16
12	42.794281	143.095111	7.56
13	42.805350	143.147889	0.60
14	42.796161	143.138389	2.00
15	42.792483	143.125611	1.12
16	42.790033	143.124389	1.72
17	42.792097	143.121000	0.87
18	42.789339	143.108778	6.48
19	42.769567	143.115167	2.48
20	42.744567	143.100111	0.28
21	42.730842	143.087278	0.60
22	42.714828	143.099444	0.60
23	42.769444	143.024986	0.68
24	42.764378	143.006611	1.12
25	42.771589	143.036167	1.08
26	42.768483	143.046278	4.24
27	42.760722	143.037306	1.16
28	42.753533	143.029000	0.56
29	42.759925	143.044278	1.92
30	42.734100	143.009125	0.32
31	42.734261	143.012792	5.76
32	42.741911	143.029667	6.04
33	42.720822	143.013431	2.68
34	42.715075	142.997833	0.80

Appendix S2. Forty-one variable sites among 76 mtDNA haplotypes of the gray-sided vole *Myodes rufocanus* occurring in the Tokachi Plain, Hokkaido, Japan. Variable sites are shown by the position from the beginning of the 412-bp sequences. Nucleotides identical to haplotype H01 are indicated by a dot.

Haplo-type	GenBank/ DDBJ accession #	Variable sites
		0 0 0 0 0 0 0 0 1 1 1 1 1 1 1 1 1 1 1 1 1 1 1 1 1 1 1 2 2 2 2 2 2 2 2 2 3 3 3 3 3
		1 3 3 3 3 4 5 6 0 0 1 2 2 3 4 4 5 5 6 6 7 7 7 8 9 9 9 0 0 1 4 6 6 6 7 8 1 2 2 2 7
		3 0 3 4 9 8 4 7 7 8 9 0 1 6 6 8 1 2 3 8 0 1 2 1 0 4 6 3 7 6 5 3 4 5 5 2 9 0 1 6 9
H01	LC745586	T A T T C A T A C C T T T T A T C C T C C A A C T A A T C A T C A C A A C A G C C
H02	LC745587	. . . . G . . . . . .
H03	LC745588	. . . . C . . . .
H04	LC745589	. . . . C T T . . . .
H05	LC745590	. . . . T T . . . .
H06	LC745591	. . . . T . . . .
H07	LC745592	. . . . T . . . . A . .
H08	LC745593	. . . . T . . . . T T . . . .
H09	LC745594	. . . . T . . . . T . . . .
H10	LC745595	. . . . T . . . . T . T . . . .
H11	LC745596	. . . . T . . . . T . . . . C . . . .
H12	LC745597	. . . . T . . . . . . T . . T . . . .
H13	LC745598	. . . . T . . . . T . . . . G . . . . . . T . . . .
H14	LC745599	. . . . T . . C . . . . . . T . . . . G . . . . . . T . . . .
H15	LC745600	. . . . T . . . . T . . . . T . . T .
H16	LC745601	. . . . T . . . . . C T . . T . G . . . . T . . . .
H17	LC745602	. . . . T . . . . . . T . . T . G . . . . T . . . .
H18	LC745603	. . . . T . . . . T . . . . . . T . . T . G . . . . T . . . .
H19	LC745604	. . . . T . . . . T . . . . T . G . . . . T . . . .
H20	LC745605	. . . . T . . . . T . G . T . . . . T . . . .
H21	LC745606	. . . . T . . . . T . G . . . . T . . . .
H22	LC745607	. . . . T . . . . T A G . . . . T . . . .
H23	LC745608	. . . C T G . . . T . . . . T . G . . . .
H24	LC745609	. . . C T . . . . T . . . . T . G . . . .
H25	LC745610	. . . C T . . . . T . . . A . . . . . T . G . . . . T . . . .

H26	LC745611	...CT....T.........T.G..............T....
H27	LC745612	...CT....T..C......T.G....................
H28	LC745613	....T....T..C......T.G....................
H29	LC745614	.........T..C.....CT.G....................
H30	LC745615	....T....T..C.....CT.G....................
H31	LC745616	.T.......T..C......T.G..C.................
H32	LC745617	.........T..C......T.G..C.................
H33	LC745618	....T....T..C......T.G..C.................
H34	LC745619	.........T..C......T.G....................
H35	LC745620	.........T..C......T.G.............G.....
H36	LC745621	.........T..C...T..T.G..............G...
H37	LC745622	.........T..C......T......................
H38	LC745623	.........T..A......T.G....................
H39	LC745624	.........T.........T.G....................
H40	LC745625	.........T.........T.G.........T.........
H41	LC745626	.........TC........T.G....................
H42	LC745627	.........T...........G............G......
H43	LC745628	.........T.........T.G............G......
H44	LC745629	.......G.T.........T.G....................
H45	LC745630	.......G.T.........T.G...G................
H46	LC745631	.........T.........T.G....G...............
H47	LC745632	.....G...T.........T.G....G...............
H48	LC745633	........TT........CT.G....G...............
H49	LC745634	.........T.........TAG....G...............
H50	LC745635	.T.......T.........TAG....G...............
H51	LC745636	.........T.........T.G.T..G...............
H52	LC745637	.........T.........TGG.T..G...............
H53	LC745638	.........T..C......T.G.T..G...............
H54	LC745639	.........T.........T.G...........T.......
H55	LC745640	.........T.........T.............T.......
H56	LC745641	.........T.........T.G...........A.......
H57	LC745642	.........T.........T.G...........G.......
H58	LC745643	....T.C..T.........T.G...........T.......

H59	LC745644	....TGC..T.........T.G........C.G........
H60	LC745645	....T....T.........T.G...........A.......
H61	LC745646	C...T....T.........T.G...................
H62	LC745647	....T....T.........T.G...................
H63	LC745648	.........T.C.......T.G...................
H64	LC745649	.........T.C.......T.....................
H65	LC745650	..C......T.........T.....................
H66	LC745651	..C......T.........T....C................
H67	LC745652	.........T.........T....C................
H68	LC745653	.........T.........T.G..C...............T
H69	LC745654	.........T.........T.G..C................
H70	LC745655	.........T.........T.G......T...........T
H71	LC745656	.........T.........T.GG.....T...........T
H72	LC745657	...................T.G......T...........T
H73	LC745658	.........T..C......T.G...G..T.......T...T
H74	LC745659	.........T..C.G....T.G...G..T.......T...T
H75	LC745660	.........T.........T.G..................T
H76	LC745661	.........T.........T....................T

Appendix S3. Examination of phylogenetic relationships among observed mtDNA haplotypes using Network 10.2.0.0 (Fluxus Technology, 2020) and the CLUSTALW Multiple Sequence Alignment Program online (https://www.genome.jp). (a) Pairwise differences among all 425-bp sequences (*N* = 673), or the mismatch distribution, calculated using Network. Observed haplotypes differed from each other by 0 to 11 sites (mean 3.063). (b) Median-joining (MJ) network (Bandelt *et al*., 1999) obtained using Network. The network conformation was too complicated to define haplogroups (i.e., groups of haplotypes sharing a common ancestor on the matriline). (c) Neighbor-joining (NJ) tree (Saitou & Nei, 1987) obtained using CLUSTALW; the topology was tested using bootstrapping (*N* = 1000). Almost all clades were not statistically supported. Bootstrap numbers over 700 are shown in red.

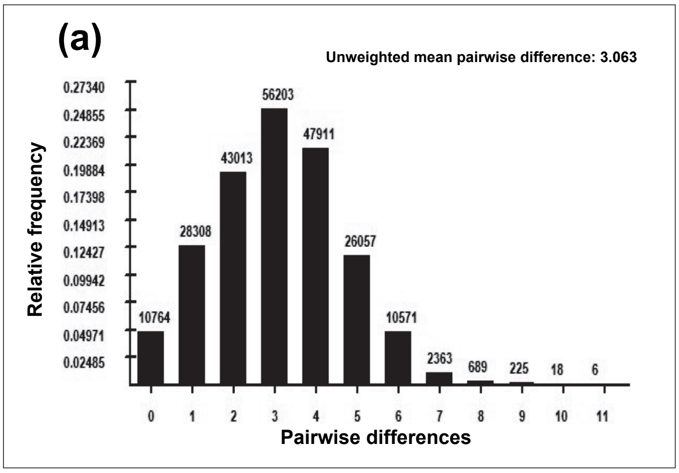

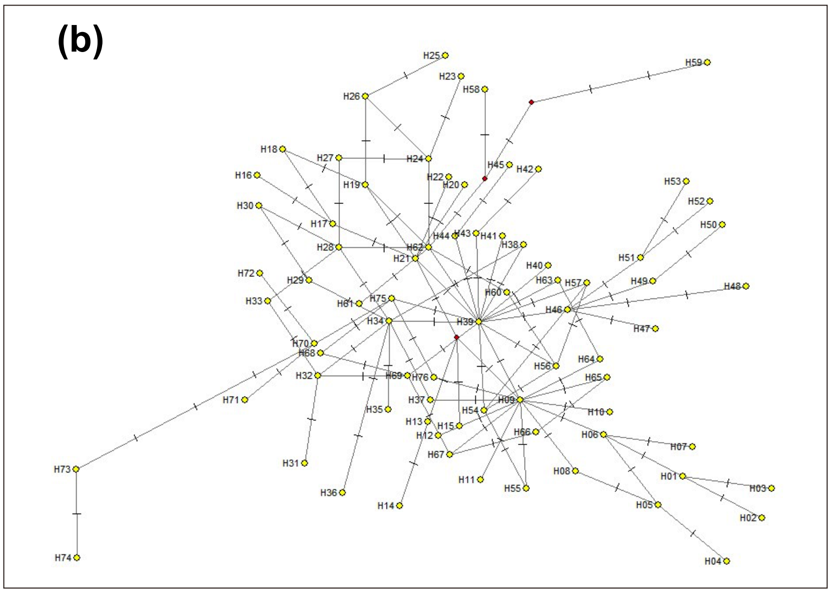

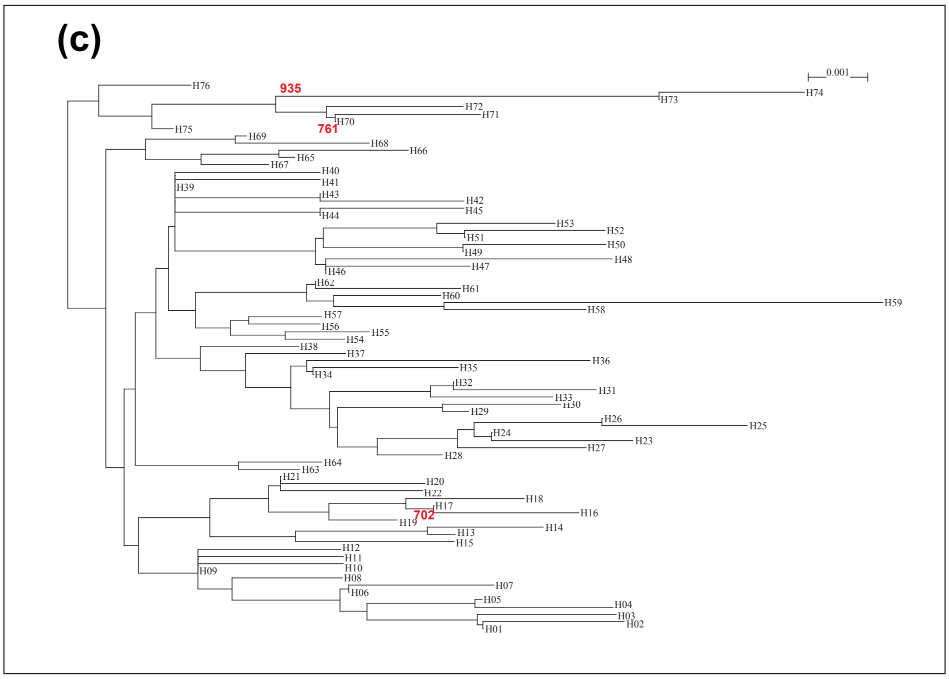

References

Bandelt, H.-J., Forster, P., & Röhl, A. (1999). Median-joining networks for inferring intraspecific phylogenies. *Molecular Biology and Evolution, 16*(1), 37–48. doi:10.1093/oxfordjournals.molbev.a026036

Saitou, N. & Nei, M. (1987). The neighbor-joining method: a new method for reconstructing phylogenetic trees. *Molecular Biology and Evolution, 4*(4), 406–425. doi:10.1093/oxfordjournals.molbev.a040454

Appendix S4. Frequency of mtDNA haplotypes at 34 trapping sites in the Tokachi Plain, Hokkaido, Japan (spring/fall).

Trapping site	Haplotype																					
	H01	H02	H03	H04	H05	H06	H07	H08	H09	H10	H11	H12	H13	H14	H15	H16	H17	H18	H19	H20	H21	H22
01																						
02	1/1		1/0								0/1									0/1		
03						1/0									1/0							0/1
04																						
05																0/1						2/2
06										2/0			2/2	4/1		1/0						
07													1/0									1/0
08	0/2																		0/1			
09																						0/1
10															0/3							
11	7/2															0/1						
12	3/0								1/0													
13								0/1	1/0										1/0			0/2
14	2/3								2/3													
15																						
16																		0/1				
17	0/1												0/1			0/1						
18			1/1						0/1				4/1			1/0		1/0				
19	0/4			1/0									1/2			0/3						
20	3/0																					
21					0/1																0/1	
22																						
23	0/1					0/1	1/6					0/2										
24																						2/7
25						2/2						0/1										
26																		0/1				
27																		1/0				
28											1/1						6/3					
29						1/0																
30		0/1		0/1																		
31																		0/3				
32									0/1									3/2				
33									2/2													
34	5/4																4/0					

Appendix S4 (continued)

Trapping site #	Haplotype H23	H24	H25	H26	H27	H28	H29	H30	H31	H32	H33	H34	H35	H36	H37	H38	H39	H40	H41	H42	H43	H44
01													1/0				3/0		2/1			
02												0/1	4/1		2/2		0/1		1/0			
03	3/0					2/5				0/1					3/5		1/5				1/0	
04										3/0												
05	0/1	1/2																				
06												2/0									1/0	
07												3/5					0/1					
08												1/1					3/0					
09																	4/4					
10		0/3		0/1					1/0			1/2					1/0					
11												1/0					2/0					
12	1/0					0/1							1/1			1/0	3/0					
13			5/0								0/1	3/4						1/0				
14	1/0										2/4				3/1		2/3					
15							1/2					2/2	0/2				1/0					
16												0/1					0/1					
17												0/2										
18												3/0					0/2					
19														1/1			2/1					1/0
20																	2/2					
21																	1/0					
22																						
23		1/0															0/3					
24												14/12										
25																	5/1					
26			5/1					1/0				0/1					1/0					
27						2/4						2/2					0/2					
28		1/0										1/0					2/5					
29												1/1				1/0	0/1			4/0		
30		1/0			4/0	1/7						1/0					1/2			1/0		
31												0/1					0/2					
32		1/1				4/3											2/8					0/1
33												0/1					0/1					
34		0/1										1/2					3/5					

Appendix S4 (continued)

Trapping site #	Haplotype																				
	H45	H46	H47	H48	H49	H50	H51	H52	H53	H54	H55	H56	H57	H58	H59	H60	H61	H62	H63	H64	H65	H66
01										1/0												
02							0/1			1/1												
03		0/1					1/0								1/0						1/0	
04		0/1										6/3										
05		1/0										1/0										
06																1/0						
07		1/0												1/0								
08												2/4		0/1								
09												2/1		5/0								
10					2/0	1/0						1/0		5/9		3/0						1/0
11		4/3					0/1					2/0		0/1					1/0			
12		1/0					1/0					4/1										1/0
13															2/0							
14																						
15							2/0													0/1		
16							0/1															
17							0/1					1/0		0/1								
18		0/1							4/1			1/4				0/1						
19								0/1								0/1	1/0					
20		2/1																				
21			0/2																			
22													3/4									
23				1/0	0/1							1/1		0/1								
24		0/1	2/0	0/1	0/1						0/1											
25		1/2						1/0				0/1						1/0				
26		1/0					1/0															
27	0/1																		1/0			1/0
28		1/1												1/0							0/1	
29		1/2																				
30				0/1																		
31		0/1											2/0	0/1							0/2	
32		2/0			1/1					1/0								0/1				
33				0/1	0/2									0/1								
34		0/1																				

Appendix S4 (continued)

Trapping site #	Haplotype										
	H67	H68	H69	H70	H71	H72	H73	H74	H75	H76	Total
01											8
02	1/0										21
03					1/0				1/0		35
04											13
05											11
06											16
07									0/2		15
08											15
09											17
10				2/0							36
11				1/3					1/0		30
12		1/0									21
13									6/7		34
14									4/0		30
15				0/1					0/1		15
16				0/1							5
17									0/3		11
18											27
19		4/5							0/1		30
20									1/0		11
21											5
22											7
23			1/1								22
24											41
25			0/1					2/0			20
26			0/1								13
27							1/1		0/1		19
28			1/2						0/1		28
29											12
30											21
31											12
32											32
33											10
34						0/3				0/1	30

Appendix S5. Characteristics of six microsatellite DNA loci in gray-sided voles captured at 18 trapping sites (n = 494).

Locus	GenBank/DDBJ accession #	Repeat motif	Size (bp)	No of alleles
MSCRB01	D37832	GT/AC	155–209	18
MSCRB04	D37835	CA/TG	68–96	18
MSCRB07	AB248755	GT/AC	105–155	24
MSCRB09	AB248757	TG/CA	135–172	18
MSCRB11	AB248759	GT/AC	192–236	21
MSCRB13	AB248760	GT/AC	115–143	16

Appendix S6. Results of the Micro-Checker analysis. The presence of null alleles was suggested at three trapping sites due to homozygote excess. The program calculates null allele frequency using four methods: Oosterhout (Van Oosterhout *et al*., 2004), Chakraborty (Chakraborty *et al*., 1992), and Brookfield 1 and 2 (Brookfield, 1996).

1. Trapping site #19 (spring)

Locus	Null Present	Oosterhout	Chakraborty	Brookfield 1	Brookfield 2
MSCRB01	no	-0.1286	-0.1101	-0.1101	0
MSCRB04	no	-0.0859	-0.0756	-0.0756	0
MSCRB07	no	-0.1037	-0.0654	-0.0621	0
MSCRB09	no	-0.1098	-0.095	-0.095	0
MSCRB11	no	-0.0068	-0.0102	-0.0092	0
MSCRB13	yes	0.17	0.1976	0.1481	0.1481

One locus shows evidence for a null allele.
This population is possibly in Hardy Weinberg equilibrium with locus MSCRB13, showing signs of a null allele.

2. Trapping site #11 (fall)

Locus	Null Present	Oosterhout	Chakraborty	Brookfield 1	Brookfield 2
MSCRB01	no	0.0213	0.0249	0.0211	0.0211
MSCRB04	no	0.0341	0.0318	0.0287	0.0287
MSCRB07	yes	0.2193	0.2763	0.1927	0.1927
MSCRB09	no	-0.1414	-0.1126	-0.1126	0
MSCRB11	no	-0.1098	-0.095	-0.095	0
MSCRB13	no	-0.0448	-0.0427	-0.0405	0

One locus shows evidence for a null allele.
This population is possibly in Hardy Weinberg equilibrium with locus MSCRB07, showing signs of a null allele.

3. Trapping site #32 (fall)

Locus	Null Present	Oosterhout	Chakraborty	Brookfield 1	Brookfield 2
MSCRB01	no	0.0431	0.0436	0.0384	0.0384
MSCRB04	yes	0.1228	0.1334	0.1096	0.1096
MSCRB07	no	-0.0881	-0.0773	-0.0773	0
MSCRB09	no	0.0139	0.0155	0.0141	0.0141
MSCRB11	no	0.0234	0.0214	0.0187	0.0187
MSCRB13	no	-0.1124	-0.09	-0.09	0

One locus shows evidence for a null allele.
This population is possibly in Hardy Weinberg equilibrium with locus MSCRB04, showing signs of a null allele.

References

Brookfield JFY (1996) A simple new method for estimating null allele frequency from heterozygote deficiency. Molecular Ecology, 5(3), 453–455.

Chakraborty R, De Andrade M, Daiger SP, Budowle B (1992) Apparent heterozygote deficiencies observed in DNA typing data and their implications in forensic applications. Annals of Human Genetics, 56(1), 45–57.

Van Oosterhout, C., Hutchinson, W. F., Wills, D. P. M., & Shipley, P. (2004). MICRO-CHECKER: software for identifying and correcting genotyping errors in microsatellite data. Molecular Ecology Notes, 4(3), 535–538.

Appendix S7. Cluster admixture proportions for 494 individuals at 18 trapping sites in two seasons under the second probable number of clusters $K = 3$. Each individual is represented by a vertical bar, and admixture proportions are denoted by different colors; clusters I, II, and III are indicated by dark blue, pale blue, and red, respectively. Numbers and letters indicate trapping sites and sampling seasons, respectively (S, spring; F, fall).

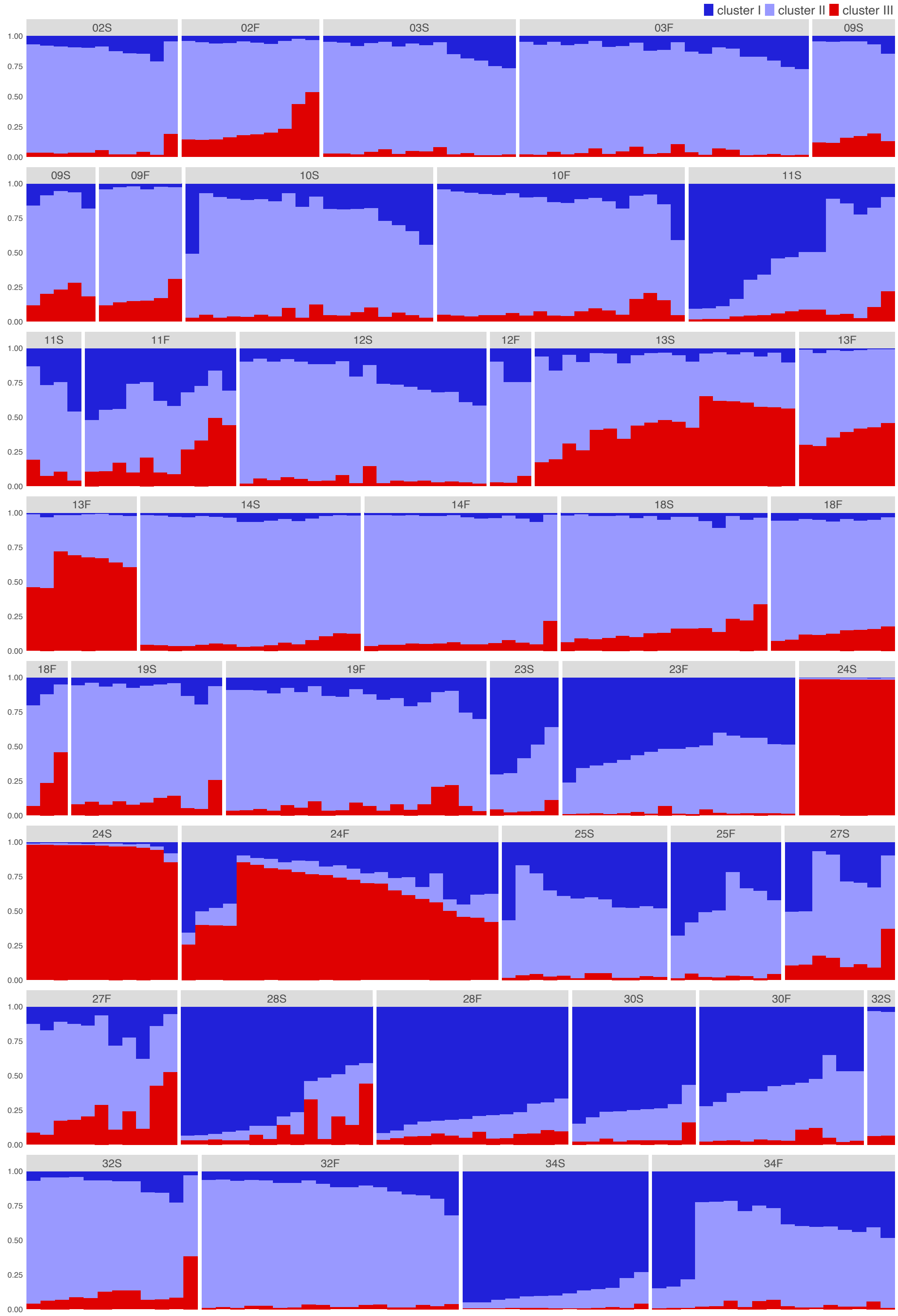

Appendix S8. Annual changes in the number of captured gray-sided voles per 150 trap-nights in August at two sites (~0.5 ha each) in Shimizu National Forest (17–31 km from the study site). Snap traps were used to capture specimens. The blue and red lines indicate the numbers caught at the native forest and afforestation sites, respectively. Arrows indicate changes in the location of trapping sites (blue and red for the native forest and afforestation sites, respectively). Until 2013, trapping sites were fixed at 17 km from the study site. The recent cycle of fluctuations appears to be getting longer.

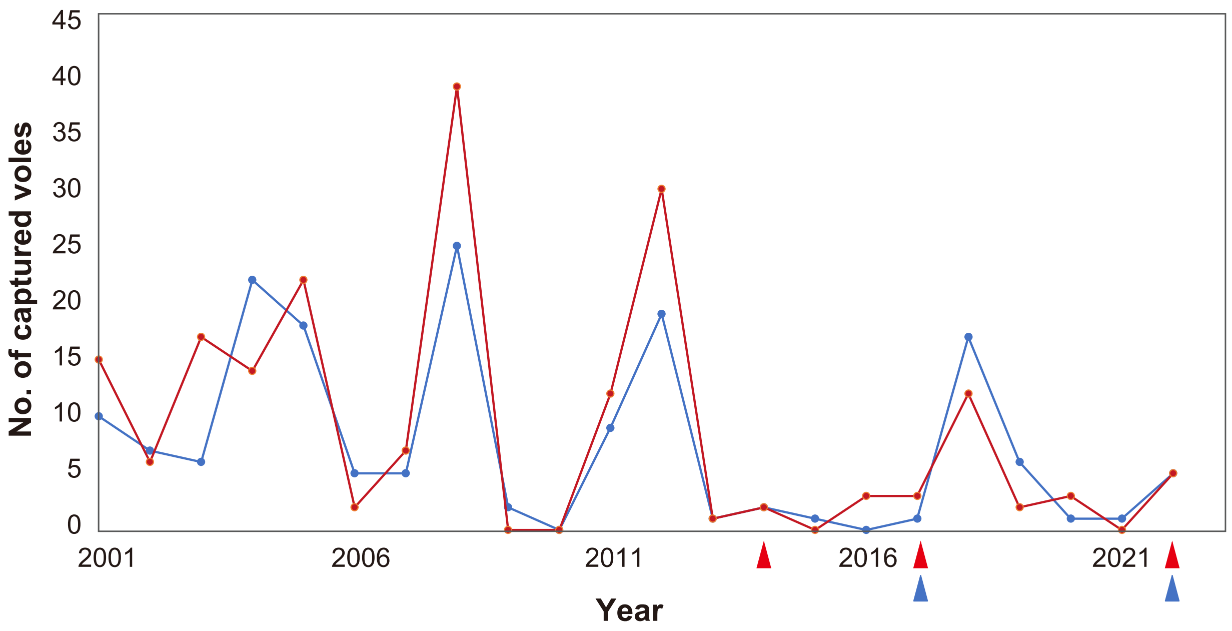

Appendix S9. Negative correlation between the mean admixture proportion of cluster I in spring and the degree of change in admixture proportions between spring and fall at $K = 3$. For each of the three clusters, a negative correlation was observed between the mean admixture proportion in spring and the degree of change in admixture proportions between spring and fall, although the correlation was not significant for cluster III (Spearman's rank-correlation test: cluster I, $S = 1432$, $P = 0.047$; cluster II, $S = 1452$, $P = 0.037$; cluster III, $S = 1092$, $P = 0.615$). Clusters I, II, and III are indicated by dark blue, pale blue, and red, respectively. Regression lines present trendlines.

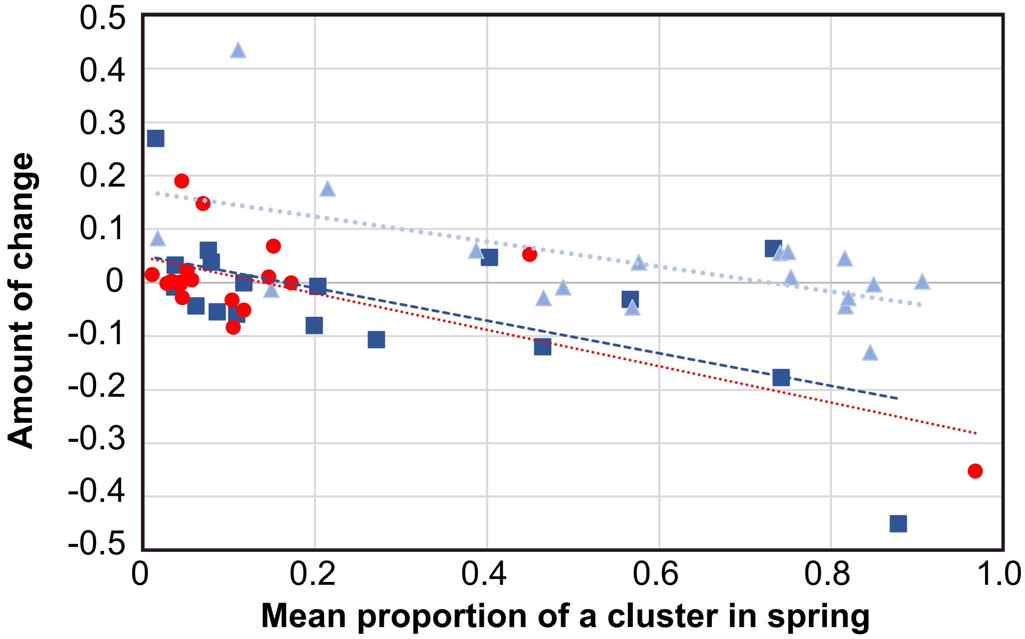

Supplement: Supplementary file 1 — Appendix S1–S9. [file ECE3-13-e10472-s001.pdf]
